# Nutrient-induced FNIP degradation by SCF^β-TRCP^ regulates FLCN complex localization and promotes renal cancer progression

**DOI:** 10.18632/oncotarget.14221

**Published:** 2016-12-25

**Authors:** Katsuyuki Nagashima, Hidefumi Fukushima, Kouhei Shimizu, Aya Yamada, Masumi Hidaka, Hisashi Hasumi, Tetsuro Ikebe, Satoshi Fukumoto, Koji Okabe, Hiroyuki Inuzuka

**Affiliations:** ^1^ Center for Advanced Stem Cell and Regenerative Research, Tohoku University Graduate School of Dentistry, Sendai 980-8575, Japan; ^2^ Department of Physiological Sciences and Molecular Biology, Fukuoka Dental College, Fukuoka 814-0193, Japan; ^3^ Department of Pathology, Beth Israel Deaconess Medical Center, Harvard Medical School, Boston, MA 02115, USA; ^4^ Division of Pediatric Dentistry, Department of Oral Health and Development Sciences, Tohoku University Graduate School of Dentistry, Sendai 980-8575, Japan; ^5^ Department of Urology and Molecular Genetics, Yokohama City University Graduate School of Medicine, Yokohama 236-0004, Japan; ^6^ Department of Oral and Maxillofacial Surgery, Fukuoka Dental College, Fukuoka 814-0193, Japan

**Keywords:** FNIP, FLCN, tumor suppressor, β-TRCP, renal cancer

## Abstract

Folliculin-interacting protein 1 and 2 (FNIP1 and FNIP2) play critical roles in preventing renal malignancy through their association with the tumor suppressor FLCN. Mutations in *FLCN* are associated with Birt-Hogg-Dubé (BHD) syndrome, a rare disorder with increased risk of renal cancer. Recent studies indicated that *FNIP1/FNIP2* double knockout mice display enlarged polycystic kidneys and renal carcinoma, which phenocopies *FLCN* knockout mice, suggesting that these two proteins function together to suppress renal cancer. However, the molecular mechanism functionally linking FNIP1/FNIP2 and FLCN remains largely elusive. Here, we demonstrated that FNIP2 protein is unstable and subjected to proteasome-dependent degradation via β-TRCP and Casein Kinase 1 (CK1)-directed ubiquitination in a nutrition-dependent manner. Degradation of FNIP2 leads to lysosomal dissociation of FLCN and subsequent lysosomal association of mTOR, which in turn promotes the proliferation of renal cancer cells. These results indicate that SCF^β-TRCP^ negatively regulates the FLCN complex by promoting FNIP degradation and provide molecular insight into the pathogenesis of BHD-associated renal cancer.

## INTRODUCTION

Mutations in the folliculin (*FLCN*) gene are responsible for Birt-Hogg-Dubé (BHD) syndrome, a rare autosomal dominant disorder characterized by cutaneous fibrofolliculoma, pulmonary and renal cysts, and bilateral renal cell carcinoma [1–3]. Single allele loss of *FLCN* is ascribed to an occurrence of the syndrome, and a mutation in the second allele or loss of heterozygosity leads to BHD-associated renal cancer development [1, 2, 4]. In mice, homozygous kidney-specific *FLCN* knockouts display enlarged polycystic kidneys [5–7], and heterozygous whole-body *FLCN* knockout mice develop renal cyst and malignancies that are reminiscent of human BHD tumors [8–10]. These studies suggest that FLCN is a critical tumor suppressor for BHD-associated renal cancer.

FLCN forms a complex with FLCN-interacting protein 1 (FNIP1) and FNIP2 [11–13] to regulate multiple cellular processes such as energy sensing [14–16], differentiation [17], autophagy [18, 19], and apoptosis [20–23]. Genetically engineered mouse models demonstrated that homozygous whole-body *FNIP1* knockout mice displayed compromised B-cell development [23–25] and altered programming of muscle fiber-type switching [26]. Whole-body *FNIP1^+/−^FNIP2^−/−^* mice develop renal cancer while kidney-specific *FNIP1^−/−^FNIP2^−/−^* mice displayed enlarged polycystic kidneys [27]. The renal phenotypes in both FNIP double knockout mouse models recapitulate the *FLCN* knockout mouse kidneys [27], suggesting a role for FNIP proteins in controlling FLCN signaling. However, the molecular mechanisms underlying the regulation of the FLCN complex are largely unknown.

Protein ubiquitination is a post-translational modification that regulates various aspects of intracellular signaling pathways [28]. Several types of enzymes and scaffolding factors are involved in the ubiquitination of target proteins, and E3 ubiquitin ligases determine target specificity [29]. Among the E3 ubiquitin ligases, SCF (Skp1-Cullin 1-F-box protein) complex is one of the most extensively characterized ubiquitin ligase complexes [30, 31]. The F-box protein β-TRCP, which comprises two distinct paralogs, β-TRCP1 and β-TRCP2, is a substrate recognition subunit of SCF^β-TRCP^. Given its critical role in regulating various signaling pathways such as the Wnt, NF-κB, and mTORC pathways, β-TRCP is considered a versatile and critical modulator in various intracellular signal transduction events [30, 32].

In this study, we investigated the molecular mechanisms underlying the regulation of FNIP proteins by post-translational modifications in a nutrient availability-dependent manner. AMPK forms a complex with, and phosphorylates FNIP1 and FNIP2, resulting in the modulation of the AMPK downstream signaling [11, 13]. Furthermore, FNIP2 protein is stabilized during apoptosis, and the proteasome inhibitor MG132 induces FNIP2 accumulation, implying the involvement of the ubiquitin-proteasome pathway in the control of FNIP2 stability [22]. Here, we elucidated the mechanisms underlying FNIP2 protein degradation and found that FNIP2 is targeted by SCF^β-TRCP^ for ubiquitination and degradation in a phosphorylation-dependent manner. Our results support a role of β-TRCP as a negative regulator of the FLCN complex.

## RESULTS

### Nutritional status controls FNIP abundance in a proteasome-dependent manner

**Figure 1:**
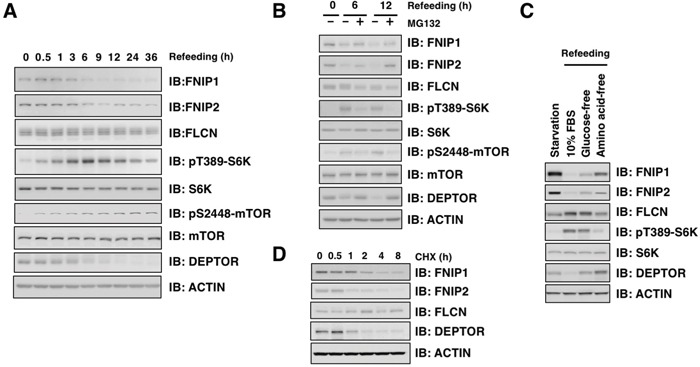
FNIP abundance is regulated by nutritional conditions **A.** HeLa cells were starved for 12 hours (h) and stimulated with fresh 10% FBS DMEM. Cells were harvested at the indicated time points and subjected to immunoblot (IB) analysis. **B.** HeLa cells were starved for 12 h and stimulated with fresh 10% FBS DMEM with or without the proteasome inhibitor MG132 (15 μM). Cells were harvested at the indicated time points and subjected to IB analysis. **C.** HeLa cells were starved for 12 h and stimulated with fresh 10% FBS, glucose-free, or amino acid-free DMEM for 6 h. Cells were harvested and subjected to IB analysis. **D.** HeLa cells were treated with 100 μg/mL cycloheximide (CHX) and harvested at the indicated time points for IB analysis.

FNIP1 and FNIP2 regulate nutrient and energy sensing through modulating mTORC1 signaling that is involved in anabolic pathways leading to cell growth [2, 33]. Thus, we sought to investigate how FNIP protein abundance is controlled by nutrient availability. FNIP protein abundance rapidly decreased upon nutrient stimulation in starved and refed HeLa cells (Figure 1A). DEPTOR, an endogenous mTORC inhibitor, similarly led to a decrease in FNIP protein, while refeeding increased mTOR and S6K phosphorylation (Figure 1A), implicating FNIP downregulation in mTORC1 activation. Given the potential role of the ubiquitin-proteasome pathway in the regulation of FNIP [22], we assessed FNIP protein abundance in starved and refed HeLa cells in the presence of MG132, a proteasome inhibitor, and found that MG132 blocked nutrient-stimulated FNIP downregulation (Figure 1B). To dissect nutrition cues that destabilized FNIP proteins, we assessed FNIP abundance in HeLa cells cultured in fresh serum-containing medium, glucose-free, or amino acid-free medium. Compared to cells cultured in fresh serum medium, FNIP protein levels modestly increased in cells cultured in glucose-free or amino acid-free medium treatment (Figure 1C). Furthermore, FNIP proteins displayed a short half-life under these growth conditions (Figure 1D). Together, these results suggest that FNIP protein stability is negatively regulated by nutrient stimulation via proteasome-dependent degradation.

### SCF^β-TRCP^ E3 ligase complex interacts with FNIP

**Figure 2:**
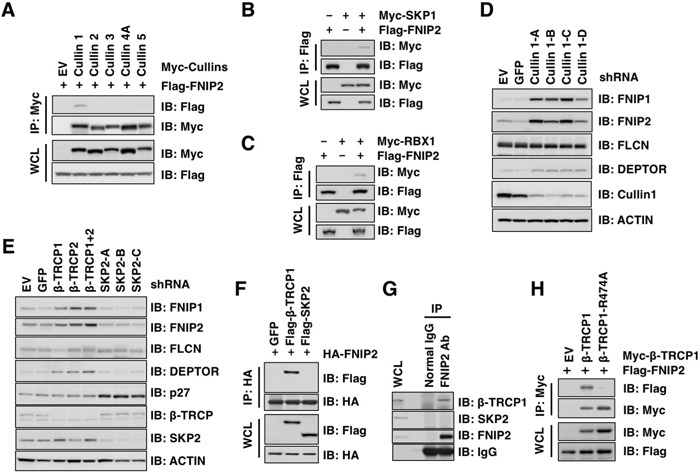
FNIP interacts with SCF^β-TRCP^ **A.** Immunoblot (IB) analysis of whole cell lysates (WCL) and Flag-immunoprecipitates (IP) derived from 293T cells transfected with Flag-FNIP2 and empty vector (EV), Myc-Cullin1, 2, 3, 4A, or 5, as indicated. At 24 h post transfection, cells were treated with 15 μM MG132 for 12 h before harvesting. **B-C.** IB analysis of WCL and Flag-IP derived from 293T cells transfected with Flag-FNIP2 and Myc-SKP1 (B) or Myc-RBX1 (C) as indicated. At 24 h post transfection, cells were treated with 15 μM MG132 for 12 h before harvesting. **D.** IB analysis of WCL derived from HeLa cells infected with lentiviral shRNA constructs against GFP and Cullin1 (four independent shRNAs namely Cullin1-A, B, C, or D) followed by selection with 1 μg/mL puromycin for 72 h to eliminate non-infected cells. **E.** IB analysis of WCL derived from HeLa cells infected with lentiviral shRNA constructs against GFP, β-TRCP1, β-TRCP2, β-TRCP1+2 (shRNA against both β-TRCP1 and β-TRCP2 paralogs), and SKP2 (three independent shRNAs namely SKP2-A, B, and C) followed by selection with 1 μg/mL puromycin for 72 h to eliminate non-infected cells. **F.** IB analysis of WCL and HA-IP derived from HeLa cells transfected with HA-FNIP2 along with empty vector (EV), Flag-β-TRCP1, or Flag-SKP2. At 24 h post transfection, cells were treated with 15 μM MG132 for 12 h before harvesting. **G.** IB analysis of WCL and FNIP2-IP derived from HeLa cells treated with MG132 for 12 h before harvesting. Endogenous interaction between FNIP2 and β-TRCP1 was confirmed. Input, 5% WCL. **H.** IB analysis of WCL and Flag-IP derived from HeLa cells transfected with Flag-FNIP2 along with empty vector (EV), Myc-β-TRCP1, or Myc-β-TRCP1-R474A. At 24 h post transfection, cells were treated with 15 μM MG132 for 12 h before harvesting.

As Cullin proteins serve as scaffolding modules in Cullin-Ring type E3 ubiquitin ligase complexes (CRL), we assessed the interactions between the various Cullin proteins and FNIP2. FNIP2 specifically interacted with Cullin 1, a scaffolding subunit of the SCF (Skp1-Cullin 1-F-box protein) complex (Figure 2A). FNIP2 also interacted with the additional SCF complex components, SKP1 and RBX1 (Figure 2B and 2C), further supporting that FNIP2 interacts with the SCF complex. Consistent with the possibility that an SCF E3 ubiquitin ligase complex destabilizes FNIP, Cullin 1 knockdown led to FNIP protein accumulation in HeLa cells (Figure 2D). Furthermore, the knockdown of either β-TRCP or SKP2, two well-characterized F-box proteins, indicated that depletion of β-TRCP1, but not that of SKP2, led to an increase in FNIP protein levels without affecting its mRNA expression levels (Figures 2E and Supplementary Figure S1A). Consistently, β-TRCP, but not SKP2, specifically bound to exogenous and endogenous FNIP2 (Figure 2F-2G). Moreover, the β-TRCP1-R474A mutant that cannot recognize its substrates [36] failed to interact with FNIP2 (Figure 2H) and FNIP1 (Supplementary Figure S1B). Taken together, these data indicate that SCF^β-TRCP^ interacts with FNIP1/FNIP2 to control their stability. Given that our results suggest that both FNIP1 and FNIP2 stability are regulated by SCF^β-TRCP^, we primarily focused on FNIP2 degradation mechanisms for the remainder of the study.

### SCF^β-TRCP^ facilitates FNIP2 degradation in a phosphorylation-dependent manner

**Figure 3:**
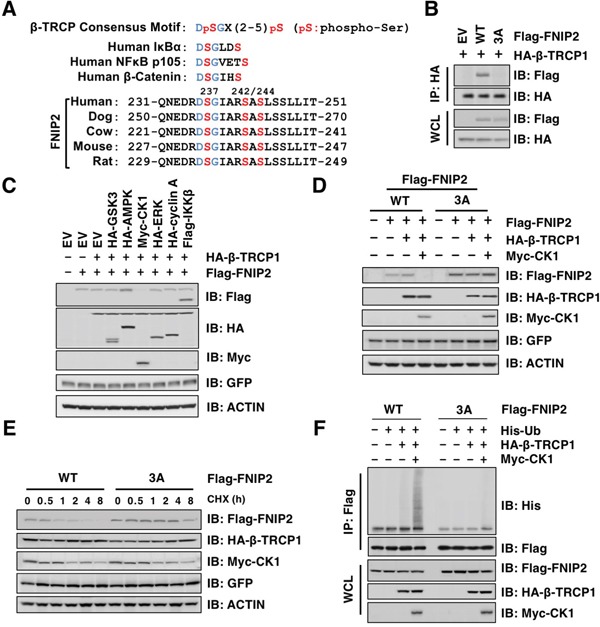
Casein Kinase 1 (CK1) promotes FNIP2 ubiquitination and degradation **A.** Alignment of FNIP2 sequences surrounding a putative β-TRCP degron motif from different species. **B.** Immunoblot (IB) analysis of whole cell lysates (WCL) and HA-immunoprecipitates (IP) derived from HeLa cells transfected with HA-β-TRCP1 along with empty-vector (EV) or the indicated Flag-FNIP2 constructs (WT or 3A: S237/242/244A). At 24 h post-transfection, cells were treated with MG132 for 12 h before harvesting. **C.** IB analysis of WCL derived from 293T cells transfected with HA-β-TRCP1 and Flag-FNIP2 along with each expression plasmids of protein kinases as indicated. GFP was included as the internal control for transfection efficiency. **D.** IB analysis of WCL derived from HeLa cells transfected with EV, Flag-FNIP2 (WT or 3A: S237/242/244A), HA-β-TRCP1, and Myc-CK1 as indicated. GFP was included as the internal control for transfection efficiency. **E.** IB analysis of WCL derived from HeLa cells transfected with Flag-FNIP2 (WT or 3A: S237/242/244A), HA-β-TRCP1, and Myc-CK1 as indicated. At 48 h post-transfection, cells were treated with 100 μg/mL cycloheximide (CHX) and harvested at the indicated time points. GFP was included as the internal control for transfection efficiency. **F.** IB analysis of WCL and anti-Flag IP derived from 293T cells transfected with EV, Flag-FNIP2 (WT or 3A: S237/242/244A), His-Ub, HA-β-TRCP1, and Myc-CK1 as indicated. At 24 h post-transfection, cells were treated with MG132 (15 μM) for 12 h before harvesting.

Prior phosphorylation of the consensus β-TRCP binding motif (phospho-degron) is critical for the SCF^β-TRCP^ complex to recognize and ubiquitinate its substrates [37]. We identified a potential β-TRCP phospho-degron motif in FNIP1 (Supplementary Figure S1C) and FNIP2 (Figure 3A) and generated a mutant in which the Ser residues within the degron motif are substituted to Ala (S237/242/244A namely 3A) for subsequent analyses (Figure 3A). The FNIP2-3A mutant was unable to interact with β-TRCP (Figure 3B), indicating that β-TRCP recognizes FNIP2 through this specific consensus degron motif. To identify a protein kinase responsible for the Ser phosphorylation in the β-TRCP degron motif, we evaluated FNIP2 protein levels following co-expression with various kinases. Of the kinases tested, expression of Casein Kinase 1 (CK1) led to a decline in FNIP2 (Figure 3C). Furthermore, the FNIP2-3A-mutant was resistant to CK1-mediated FNIP2 downregulation (Figure 3D), and the half-life of FNIP2-3A was largely extended compared to WT FNIP2 (Figure 3E). Consistent with a role for CK1 in regulating FNIP2 stability, co-expression of CK1 and β-TRCP enhanced FNIP2 poly-ubiquitination (Figure 3F). Together, these results indicate that CK1-mediated phosphorylation promoted SCF^β-TRCP^-dependent ubiquitination and subsequent degradation of FNIP2.

### SCF^β-TRCP^ regulates FLCN and mTOR lysosomal localization

**Figure 4:**
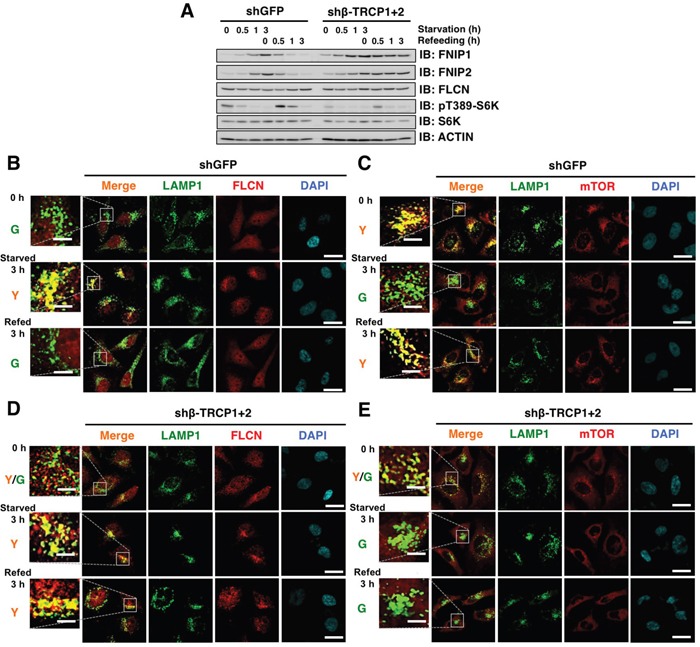
β-TRCP knockdown leads to FNIP stabilization, increased FLCN lysosomal localization, and diffused mTOR distribution in HeLa cells **A.** Immunoblot (IB) analysis of whole cell lysates derived from HeLa cells stably expressing shRNA against GFP (control) or β-TRCP1+2 (shRNA against both β-TRCP1 and β-TRCP2). After being serum and amino acid-starved for 3 h, cells were treated with fresh 10% FBS DMEM and harvested at the indicated time points. **B-E.** Confocal images of HeLa cells presented in (A). DAPI-loaded HeLa cells were analyzed for co-localization of FLCN (B and D) (red) or mTOR (C and E) (red) with a lysosomal marker, LAMP1 (green). Y (yellow) indicates predominant localization of FLCN or mTOR in the lysosome. Scale bars, 20 μm (5 μm in the enlarged images).

To further evaluate the contribution of SCF^β-TRCP^ activity towards nutrient-dependent degradation of FNIP, we depleted *β-TRCP* in HeLa cells and examined FNIP protein levels under starved and refed conditions. Following starvation, FNIP protein accumulated in both control and *β-TRCP* knocked down cells (Figure 4A). However, upon nutrient stimulation, FNIP protein levels decreased in control cells, while *β-TRCP* depleted cells sustained higher levels of FNIP, in spite of the decrease in *FNIP* mRNA expression (Supplementary Figure S2A and S2B). These data suggest that β-TRCP is involved in the degradation of FNIP in a nutrient-dependent manner.

Starvation evokes FLCN recruitment to lysosomes, and FNIP co-expression facilitates the FLCN accumulation at the lysosomal membrane through the formation of the FLCN complex [14–16]. Therefore, we assessed how *β-TRCP* depletion influenced FLCN distribution in a nutrient-dependent manner. Under growth conditions, no co-localization was observed between FLCN and a lysosomal marker, LAMP1 (Figure 4B). However, after 3 h of starvation, FLCN was associated with lysosomes in the perinuclear region, and refeeding promoted the dissociation of FLCN from the lysosomal membrane (Figure 4B). The lysosome is an intracellular organelle where mTORC1 complex is recruited and activated in a nutrition availability-dependent manner [33, 38]. Consistently, lysosome-associated mTOR relocated to the cytoplasm following starvation, and lysosomal association of mTOR was quickly restored when cells were placed back in nutrient-rich conditions, (Figure 4C). *β-TRCP* depletion led to a partial enrichment of FLCN to the lysosomal membrane under growth conditions, and enhanced enrichment of FLCN to lysosomes was retained even 3 h post nutrition stimulation (Figure 4D), whereas mTOR continued to be diffusely localized in the cytoplasm (Figure 4E). These results suggest a causal relationship of lysosomal localization between FLCN and mTOR complexes.

**Figure 5:**
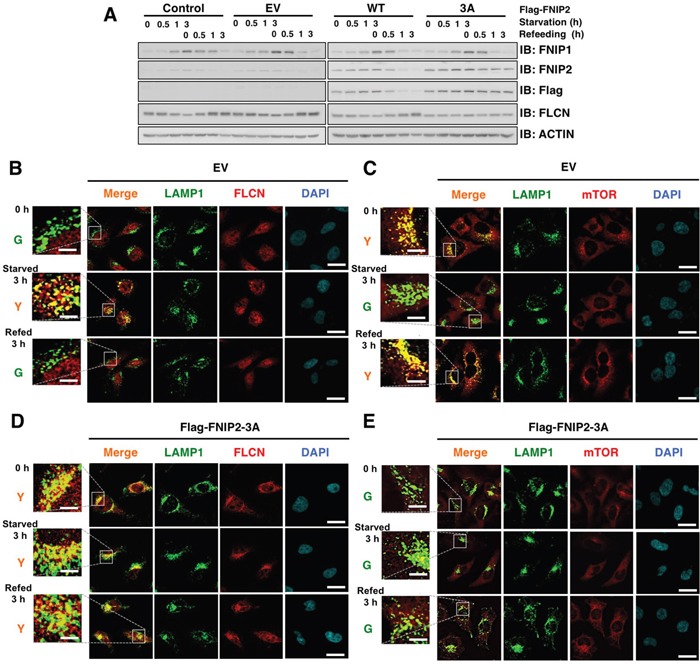
Ectopic expression of non-degradable FNIP2 enhances FLCN lysosomal localization and diffusion of mTOR in HeLa cells **A.** Immunoblot (IB) analysis of whole cell lysates derived from HeLa cells transfected with empty vector (EV) or FNIP2-expression plasmid (WT or 3A: S237/242/244A). At 12 h post-transfection, cells were treated with 0.4 mg/mL G418 for 72 h to eliminate non-transfected cells. After being serum and amino acid-starved for 3 h, cells were treated with fresh 10% FBS DMEM and harvested at the indicated time points. **B-E.** Confocal images of HeLa cells presented in (A). DAPI-loaded HeLa cells were analyzed for co-localization of FLCN (B and D) (red) or mTOR (C and E) (red) with a lysosomal marker, LAMP1 (green). Y (yellow) indicates predominant localization of FLCN or mTOR in the lysosome. Scale bars, 20 μm (5 μm in the enlarged images).

**Figure 6:**
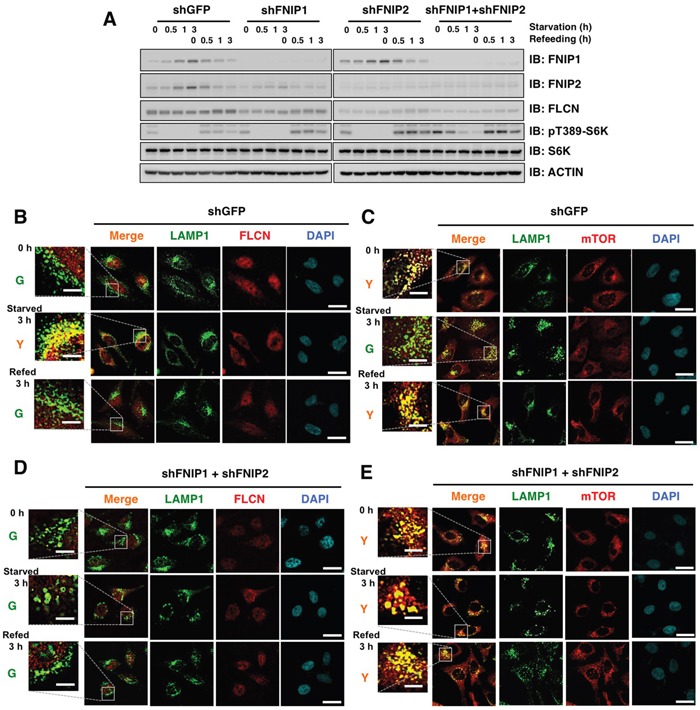
FNIP knockdown results in increased mTOR lysosomal localization and diffusion of FLCN in HeLa cells **A.** Immunoblot (IB) analysis of whole cell lysates derived from HeLa cells stably expressing shRNA against GFP (control), FNIP1, FNIP2, or FNIP1/FNIP2. After being serum and amino acid-starved for 3 h, cells were treated with fresh 10% FBS DMEM and harvested at the indicated time points. **B-E.** Confocal images of HeLa cells presented in (A). DAPI-loaded HeLa cells were analyzed for co-localization of FLCN (B and D) (red) or mTOR (C and E) (red) with a lysosomal marker, LAMP1 (green). Y (yellow) indicates predominant localization of FLCN or mTOR in the lysosome. Scale bars, 20 μm (5 μm in the enlarged images).

Given the possible pleiotropic effects *of β-TRCP* knockdown, we ectopically expressed Flag-FNIP2 WT and non-degradable FNIP2-3A mutant in HeLa cells to verify specific roles of β-TRCP/FNIP signaling in controlling the FLCN and mTOR pathways. While FNIP2-WT was promptly degraded following nutrient stimulation, FNIP2-3A mutant was unchanged (Figure 5A). Immunofluorescence analyses revealed that FLCN continued to reside on the lysosomal membrane in Flag-FNIP2-3A expressing cells, while dispersion of mTOR from lysosomes was observed regardless of the nutrient status (Figure 5B-5E). To further validate the role of FNIP in the process (Figure 6A), we depleted *FNIP1/FNIP2*, and examined the lysosomal localization of FLCN and mTOR. Knockdown of *FNIP* led to a nutrient-independent lysosomal localization of mTOR with an increase in pT389-S6K, while it abrogated a starvation-induced lysosome association of FLCN (Figure 6A-6E). Collectively, these data indicated that FNIP abundance is a critical determinant of lysosomal localization of both FLCN and mTOR, suggesting that β-TRCP-mediated FNIP degradation activates mTORC1 signaling in a nutrient-dependent manner.

### The FLCN complex suppresses proliferation and tumorigenic capacity of UOK-257 renal cancer cells

FLCN-null UOK-257 cells are an epithelial renal carcinoma line derived from a patient with BHD syndrome, and UOK-257-2 cells were generated as a counterpart cell line by re-introducing FLCN in UOK-257 cells [11]. Since β-TRCP-mediated FNIP degradation facilitates mTOR lysosomal enrichment, we evaluated FNIP levels and mTORC1 activity in the starved and refed UOK-257 cells. Consistent with a previous report [11], loss of FLCN led to activated mTORC1 signaling presumably due to the absence of the FLCN complex (Supplementary Figure S3A). As expected, loss of FLCN displayed mTOR enrichment to the lysosomal outer membrane, independently of nutrient availability (Supplementary Figure S3B-S3C). Conversely, FLCN restoration in UOK-257-2 cells and its association with lysosomes caused the dissociation of mTOR from lysosomes in a starvation-dependent manner (Supplementary Figure S3D-S3E).

**Figure 7:**
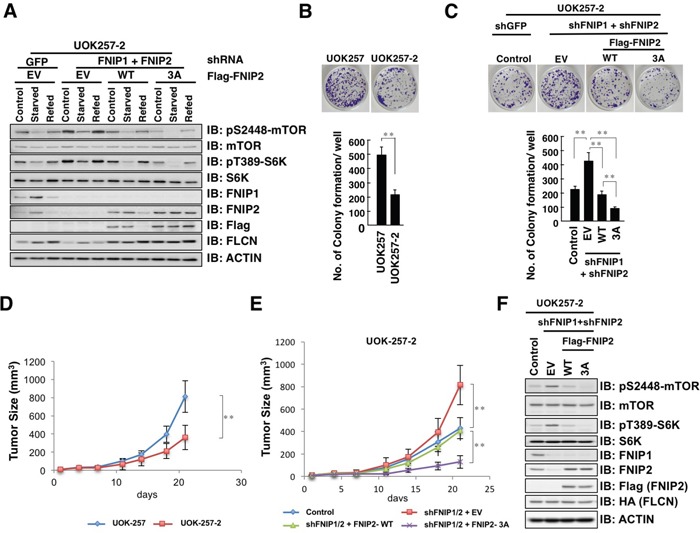
Non-degradable FNIP2 mutant significantly suppresses the proliferative and tumorigenic capacity of UOK-257-2 cells **A.** Immunoblot (IB) analysis of whole cell lysates derived from UOK-257-2 cells infected with shRNA lentiviral plasmids against FNIP1 and FNIP2 followed by selection in hygromycin and puromycin containing medium for 72 h, and subsequently transfected with empty vector (EV) or Flag-FNIP2 expression plasmids (WT or 3A: S237/242/244A). At 12 h post-transfection, cells were treated with 0.4 mg/mL G418 for 7 days to eliminate non-transfected cells. After being serum-starved for 3 h, cells were treated with fresh 10% FBS DMEM for 3 h before harvesting. **B.** Clonogenic colony formation of UOK-257 and UOK-257-2 cells (upper panel) was quantified (lower panel). Data are presented as means ± SD, n = 3, ** p < 0.01. **C.** Clonogenic colony formation of UOK-257-2 cells presented in Figure 7A (upper panel) was quantified (lower panel). Data are presented as means ± SD, n = 3, ** p < 0.01. **D.** Growth curve of developed tumors derived from xenografted UOK-257 or UOK-257-2 cells. The indicated tumor cells (3 × 106) were subcutaneously inoculated into each flank of 6 nude mice. The size of visible tumors was measured at the indicated days post-injection. Data are presented as means ± SD, n = 6, ** p < 0.01. **E.** Growth curve of developed tumors derived from xenografted UOK-257-2 cells presented in Figure 7A or 7C. The indicated tumor cells (3 × 106) were subcutaneously inoculated into each flank of 6 nude mice. The size of visible tumors was measured at the indicated days post-injection. Data are presented as mean ± SD, n = 6, ** p < 0.01. **F.** IB analysis of whole cell lysates derived from dissected solid tumors presented in E.

To investigate the role of β-TRCP-mediated FNIP degradation in renal cancer cell growth, we manipulated UOK-257-2 cells by depleting *FNIP1/FNIP2* and reintroducing FNIP2-WT or non-degradable FNIP2-3A. Consistent with studies using *FNIP1/FNIP2* double knockout mice [26], *FNIP* knockdown in UOK-257-2 cells led to the activation of mTORC1 signaling (Figure 7A). Conversely, reintroducing FNIP2-WT in the *FNIP*-depleted cells diminished the enhanced mTORC1 activity, whereas reintroducing the non-degradable FNIP2-3A mutant further enhanced mTORC1 activity (Figure 7A). Furthermore, FLCN expression suppressed colony formation (Figure 7B), while the FLCN-dependent anti-proliferative effects were reversed by *FNIP* depletion in UOK-257-2 cells (Figure 7C). Reintroducing FNIP2-WT conversely blocked colony formation of the *FNIP*-depleted UOK-257-2 cells whereas reintroducing the FNIP2-3A mutant further suppressed cell growth, suggesting a tumor suppressive role of FNIP2 and a contribution of β-TRCP in promoting renal cancer cell proliferation (Figure 7C). In addition, consistent with a previous report [39], FLCN restoration suppressed cell migration using an *in vitro* wound healing assay (Supplementary Figure S4A). Furthermore, *FNIP* knockdown facilitated the migration of UOK-257-2 cells, while reintroducing FNIP2-WT slowed cell migration, which was further retarded by FNIP2-3A expression (Supplementary Figure S4B).

To validate our *in vitro* studies, we performed mouse xenograft experiments using UOK-257 and UOK-257-2 cells. Consistent with a tumor suppressor function of FLCN, tumor growth of UOK-257-2 cells, which express FLCN, was significantly suppressed when compared to that of FLCN-null UOK-257 cells (Figure 7D). Furthermore, *FNIP1/FNIP2* depletion enhanced tumorigenesis (Figure 7E). Reintroducing FNIP2-WT in *FNIP1/FNIP2* depleted cells resulted in decreased tumor size and FNIP2-3A mutant presented further suppressive effects (Figure 7E). Consistent with *in vitro* studies, mTORC1 activity was inversely correlated with FNIP2 abundance in xenografts (Figure 7F). Taken together, these data indicate a critical tumor suppressive role of the FLCN complex in BHD-associated renal cancer proliferation and tumorigenesis, and β-TRCP counteracts the observed tumor suppressive function through FNIP degradation.

## DISCUSSION

Birt-Hogg-Dubé (BHD) syndrome, caused by mutations in the *FLCN* gene, is a rare autosomal dominant disorder with an increased incidence in renal cell carcinoma [1–3]. FLCN forms a complex with FNIP1 and FNIP2 [11–13] to regulate multiple cellular processes, including energy sensing [14–16], differentiation [17], autophagy [18, 19], and apoptosis [20–23]. However, how this complex senses nutrient availability to control renal cancer tumorigenesis has remained elusive. In this study, we identified that nutrient-sensitive proteasome-dependent FNIP degradation promoted renal cancer development by suppressing the formation of the FLCN complex. FNIP degradation facilitated the replacement of FLCN by mTOR on the lysosomal surface, which, in turn, activated mTORC1 signaling associated with renal cancer development and progression [40, 41]. Notably, *FNIP1/FNIP2* double knockdown promoted cell proliferation and migration, which were suppressed by reintroducing the non-degradable FNIP2 mutant, validating the critical tumor suppressor role of FNIP proteins.

We further identified that nutrient stimulation promotes FNIP2 degradation in a β-TRCP-dependent manner. Yet, how changes in nutrient status direct β-TRCP-mediated degradation of FNIP2 remains unclear. Interactions between β-TRCP and its substrates are regulated in a phosphorylation-dependent manner and given that CK1 is required to phosphorylate FNIP2, thereby promoting its recognition by β-TRCP, it is plausible that CK1-mediated FNIP phosphorylation is induced in a nutrient stimuli-dependent manner. While CK1 activation mechanisms are not well characterized, phosphorylation of CK1 C-terminal regulatory domain may be involved in modulating CK1 activity [42]. For instance, Ser370 in rat CK1δ C-terminal domain is subjected to PKA, PKC, or Akt-mediated phosphorylation, thereby increasing CK1 kinase activity, suggesting that CK1 S370 phosphorylation is a potential target for nutrient stimulated signaling. Alternatively, CK1 often requires priming phosphorylation in consensus CK1 recognition motifs on target proteins [43]. For instance, the transcription factor, Gli3, is initially phosphorylated by PKA, which primes Gli3 for subsequent phosphorylation by CK1 to induce β-TRCP interaction [44, 45]. Likewise, β-TRCP-directed DEPTOR ubiquitination requires priming phosphorylation by mTOR prior to CK1-directed phosphorylation [46, 47]. Therefore, regulation of the kinase targeting sites that prime FNIP2 for CK1-mediated phosphorylation could be another mechanism driving nutrition-dependent FNIP2 degradation.

Previous studies suggest that β-TRCP induces mTORC signaling through degradation of DEPTOR and PHLPP1 [46–49]. We identified that SCF^β-TRCP^ degrades the mTORC-signaling modulator, FNIP2 (and FNIP1), to enhance mTORC1 activity. Although the molecular mechanisms by which FNIP degradation results in mTORC1 activation remain unclear, we hypothesize that the FLCN complex and mTORC1 compete for binding to Rag proteins on the lysosomal surface. Nutrient stimulation facilitates the interaction between mTORC1 and Rag proteins due to the absence of the FLCN complex, while starvation-induced FNIP accumulation facilitates the replacement of lysosome-associated mTORC1 by the FLCN complex, thereby downregulating mTORC1 activity. Our study confirmed the critical role of β-TRCP as a key regulator of mTORC1 signaling. β-TRCP promotes the degradation of FNIP and DEPTOR to activate the amino acid-dependent Rag pathway and growth factor-dependent Rheb pathway respectively, both of which are required for full activation of mTORC1 [50]. Given the frequent observation of mTORC1 activation in the genetically engineered *FLCN* or *FNIP*-null mouse models [5–7, 9, 24, 27] as well as in patients with BHD [51], our results provide further support for the use of mTOR inhibitors for patients with BHD syndrome, which has been evaluated in both *in vitro* [52] and *in vivo* models [5, 7, 53] and is currently being evaluated in a clinical trial (NCT02504892).

Our results highlight the molecular mechanisms and physiological significance of SCF^β-TRCP^-mediated FNIP degradation. We identified that FNIP protein abundance is inversely correlated with the tumorigenic capacity of BHD-associated renal cancer cells. Thus, our finding suggests that the β-TRCP and CK1 signaling, responsible for nutrient-dependent degradation of FNIP, is a potential therapeutic target in renal cancer.

## MATERIALS AND METHODS

### Cell culture and transfection

HeLa and 293T cell lines (ATCC) were cultured in DMEM containing 10% FBS and antibiotics (100 μg/mL streptomycin and 100 units penicillin). The *FLCN*-null UOK-257 and *FLCN*-restored UOK-257-2 human clear-cell renal cancer cell lines were obtained from Dr. Laura S. Schmidt (National Cancer Institute, Bethesda, MD, USA) and cultured as described previously [11, 54]. HeLa and 293T cells were transfected with Lipofectamine (Invitrogen, Carlsbad, CA, USA) in Opti-MEM (Invitrogen) according to the manufacturer's instructions. For the half-life experiment, transfected cells were split into 60-mm tissue culture dishes at 24 h after transfection, and treated with 100 μg/mL cycloheximide (Sigma-Aldrich, St Louis, MO, USA) the next day. At indicated time points, cells were harvested for western blot analysis. Packaging of lentiviruses, retroviruses, and subsequent infections of various cell lines were performed as described previously [55]. Where indicated, HeLa, UOK-257, or UOK-257-2 cells were starved in Earle's Balanced Salt solution (Sigma-Aldrich) for 3 h and stimulated with 10% FBS, glucose-free or glutamine-free DMEM (Gibco) for a subsequent western blot or immunofluorescence analysis.

### Plasmids

Flag-FNIP2 expression plasmid was described previously [22]. FNIP2 mutants were generated with QuikChange XL Site-Directed Mutagenesis kit (Agilent Technologies, Santa Clara, CA, USA) according to the manufacturer's instruction. Short hairpin RNA (shRNA) lentiviral pLKO vectors against β-TRCP1, β-TRCP2, β-TRCP1+2, GFP, and the HA-β-TRCP1 expression plasmid were described previously [47]. Lentiviral shRNA plasmid pLKO-hygro-shFNIP1 and pLKO-puro-shFNIP2 were purchased from Sigma-Aldrich. Myc-β-TRCP1, Myc-β-TRCP1-R474A, Flag-β-TRCP1, Myc-SKP1, Myc-RBX1, and shRNA lentiviral vectors against Cullin-1 were obtained from Dr. J. Wade Harper (Harvard Medical School, Boston, MA, USA). HA-GSK3β, HA-ERK, HA-CyclinA, Flag-IKKβ, Myc-CKI, and Myc-Cullins were described previously [56]. Flag-SKP2 plasmid and shRNA lentiviral pLKO vectors against SKP2 were described previously [55].

### Antibodies and reagents

Anti-β-TRCP1 (4394), anti-AKT (2920), anti-phospho-AKT (4060), anti-mTOR L27D4 (4517), anti-mTOR 7C10 (2983), anti-phospho-mTOR (5536), anti-S6K (2708P), anti-phospho-S6K (9234P), anti-DEPTOR (11816), anti-FLCN D14G9 (3697), and anti-GFP (2955) antibodies were purchased from Cell Signaling Technology (Danvers, MA, USA). Anti-c-Myc 9E10 (sc-40), rabbit polyclonal anti-HA (sc-805), anti-p27 (sc-528), anti-SKP2 (sc-74477), anti-Actin (sc-47778), and anti-FLCN FL-342 (sc-25777) antibodies were purchased from Santa Cruz Biotechnology (Santa Cruz, CA, USA). Mouse monoclonal anti-Flag antibody (F-1804), peroxidase-conjugated anti-mouse secondary antibody (A-4416), peroxidase-conjugated anti-rabbit secondary antibody (A-4914), and anti-Flag agarose beads (A-2220) were purchased from Sigma-Aldrich. Monoclonal anti-HA antibody (MMS-101P) was purchased from Covance (Princeton, NJ, USA). Anti-FNIP1 antibody (ab134969) was purchased from Abcam (Cambridge, MA, USA). Anti-FNIP2 antibody (HPA042779) was purchased from Atlas Antibodies AB (Stockholm, Sweden).

### Immunoblots and immunoprecipitation

Cells were lysed in a cell lysis buffer (50 mM Tris pH 7.5, 120 mM NaCl, and 0.5% NP-40) supplemented with protease inhibitors (Complete Mini, Roche, Basel, Switzerland) and phosphatase inhibitors (phosphatase inhibitor cocktail set I and II, Calbiochem, Billerica, MA, USA). The protein concentrations of the lysates were measured by using the Bio-Rad protein assay reagent. The lysates were resolved by SDS-PAGE and immunoblotted with the indicated antibodies. For immunoprecipitation, 1 mg of lysates was incubated with the appropriate antibody (1–2 μg) for 6 h at 4°C, followed by 1 h incubation with Protein A sepharose beads (GE Healthcare, Little Chalfont, UK). Immuno-complexes were washed five times with NETN buffer (20 mM Tris, pH 8.0, 100 mM NaCl, 1 mM EDTA, and 0.5% NP-40) before being resolved by SDS-PAGE and immunoblotted with the indicated antibodies. At 30 h post-transfection, cells were pretreated with 15 μM MG132 for 12 h to block the proteasome pathway before harvesting for immunoprecipitation experiments.

### Real-time RT-PCR

Total RNA was extracted with TRIzol Reagent (Invitrogen) and the reverse transcription reaction was performed with the Superscript III First Strand RT-PCR kit. After mixing the resulting templates with primers specific for *FNIP1, FNIP2, FLCN*, or *GAPDH* and SYBR Green PCR Master Mix (Applied Biosystems, Foster City, CA, USA), the real-time RT-PCR reaction was performed with the ABI-7500 Fast Real-time PCR system. The followings are the primers used for the quantitative real-time PCR: *GAPDH:* Forward, 5′-GGAGCGAGATCCCTCCAAAAT-3′, Reverse, 5′-GG CTGTTGTCATACTTCTCATGG-3′, *FLCN:* Forward, 5′-TCTTCAGCATTGTCCGCCAG-3′, Reverse, 5′-AGT TGATGAGGTAGATCCGGTC-3′, *FNIP1:* Forward, 5′-CCTGTCTGGCTTACAATGATGT-3′, Reverse, 5′-GCA AGATGATTGGTCAGAACTGC-3′, *FNIP2:* Forward, 5′-GGAAGGACCCGCCTTTAGTT-3′, Reverse, 5′-CTG GCAGCACTTGGCTGATA-3′.

### Immunofluorescence microscopy

HeLa, UOK-257, or UOK-257-2 cells grown on chamber slides (Iwaki, 5732-008) were fixed in 4% paraformaldehyde/0.1 M sodium phosphate buffer (pH7.2) for 30 min, permeabilized with 0.1% Triton X-100 for 5 min, and incubated in blocking buffer (1.5% goat serum albumin in PBS) for 2 h. The cells were stained with a primary antibody (anti-FLCN, mTOR, or LAMP1 at 1:200) in PBS overnight at 4°C. Cells were washed four times for 5 min with PBS and incubated with secondary Alexa Fluor 568-conjugated goat anti-rabbit antibody and Alexa Fluor 488-conjugated rabbit anti-mouse antibody (Invitrogen at 1:1000 each) for 40 min at room temperature. Cells were then rinsed four times for 5 min with PBS, stained with 4,6-diamidino-2-phenylindole (DAPI) for 3 min, and mounted in PermaFluor Aqueous Mounting Medium (Thermo Fisher, Waltham, MA, USA) with cover glasses (0.12–0.17 mm, 24 × 24 mm). The slides were examined with a confocal microscope (Fluoview FV10i; Olympus, Tokyo, Japan).

### Clonogenic colony forming assay

Each cell line was plated at 500 cells in 60-mm culture dishes with fresh culture medium in triplicate. The medium was replaced every 3 days. One week later, the cells were washed 3 times with PBS and fixed with 4% paraformaldehyde for 15 min. Formed colonies were stained with 0.4% crystal violet (Sigma-Aldrich) and visible colonies were counted.

### *In vitro* wound healing assay

UOK-257 or UOK-257-2 cells were cultured in 6-well plate until the cells grew to confluence. The scratch wound was scraped in a straight line using a pipette tip. Photographic images were taken at 0 h and 24 h. Gap area measurements were conducted with Image J. Percent of wound healing area was calculated by rate of wounded area between 0 and 24 h.

### Tumor xenograft assay

For tumor xenograft assay, 3 × 10^6^ of UOK-257 or UOK-257-2 cells were mixed with matrigel (1:1) and injected into the flanks of nude mice. Tumor size was measured every three or four days with calipers, and the tumor volume was determined with the formula: L × W^2^ × 0.52, where L is the longest diameter and W is the shortest diameter. Mice were euthanized at 21 days post injection. Xenografted tumors were dissected out and lysed in EBC buffer for western blot analysis to evaluate phosphorylation status of S6K and mTOR. All animal experiments complied with the Institute of Laboratory Animal Research Guide for the Care and Use of Laboratory Animals and approved by the University Committee on Use and Care of Animals at the Tohoku University.

### Statistical analysis

Data shown are representative of three to six independent experiments. All quantitative data are presented as the mean ± SD of at least three independent experiments and were analyzed by Student's *t* test for between group differences. *p* < 0.05 was considered statistically significant.

## SUPPLEMENTARY FIGURES


