# Supplementary material for: Nutrient-induced FNIP degradation by SCFβ-TRCP regulates FLCN complex localization and promotes renal cancer progression
Source: Oncotarget. 2016 Dec 25;8(6):9947–60. doi: 10.18632/oncotarget.14221 (PMC5354783; doi:10.18632/oncotarget.14221)
Supplement: Supplementary file 1 [file oncotarget-08-9947-s001.pdf]

Nutrient-induced FNIP degradation by SCF$^{β\text{-}TRCP}$ regulates FLCN complex localization and promotes renal cancer progression

SUPPLEMENTARY FIGURES

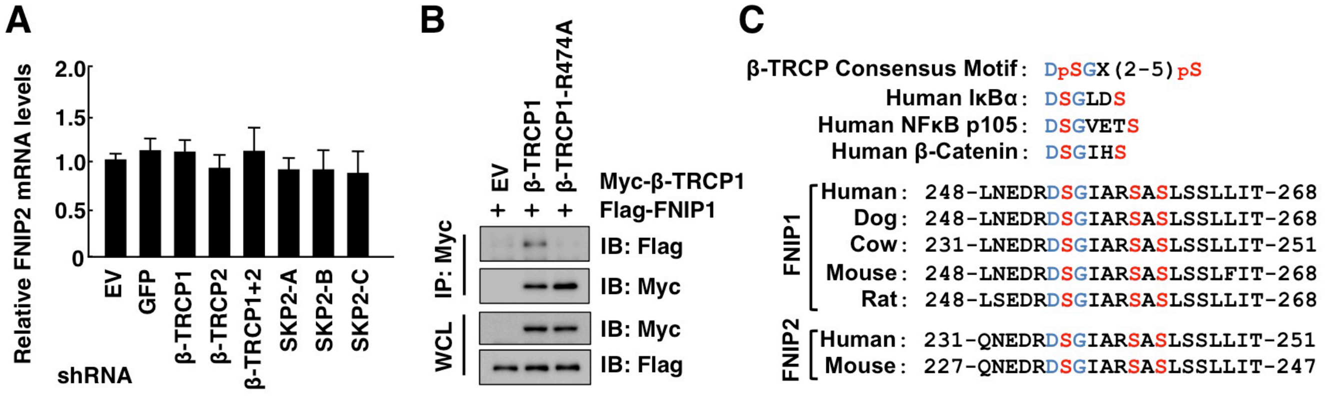

Supplementary Figure 1: SCF$^{β\text{-}TRCP}$ interacts with FNIP proteins. A. Quantitative real-time RT-PCR measuring *FNIP2* mRNA expression levels in HeLa cells presented in Figure 2E. *GAPDH* was used as an internal control. Data represent mean ± SD, n = 3. **B.** Immunoblot (IB) analysis of whole cell lysates (WCL) and Myc-immunoprecipitates (IP) derived from HeLa cells transfected with Flag-FNIP1 along with empty vector (EV), Myc-β-TRCP1 or Myc-β-TRCP1-R474A. At 24 h post transfection, cells were treated with 15 µM MG132 for 12 h before harvesting. **C.** Alignment of FNIP1 sequences surrounding a putative β-TRCP degron motif from different species and FNIP2.

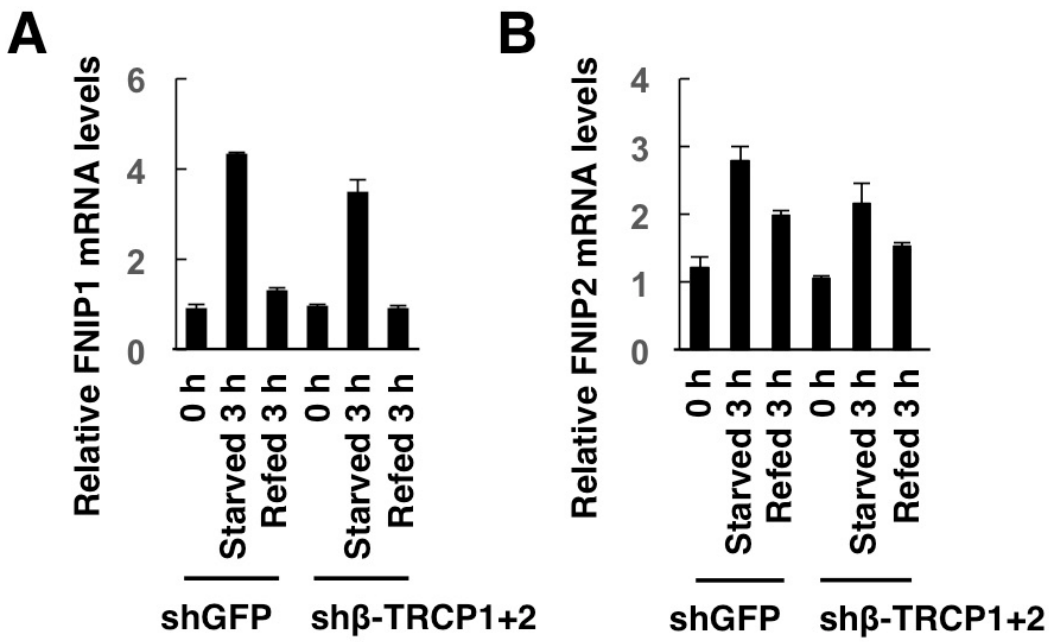

Supplementary Figure 2: FNIP abundance are regulated at transcriptional and post-translational levels. Quantitative real-time RT-PCR analysis measuring relative mRNA levels of *FNIP1* **A.** and *FNIP2* **B.** in HeLa cells presented in Figure 4A. *GAPDH* was used as an internal control. Data are represented as mean ± SD, n = 3.

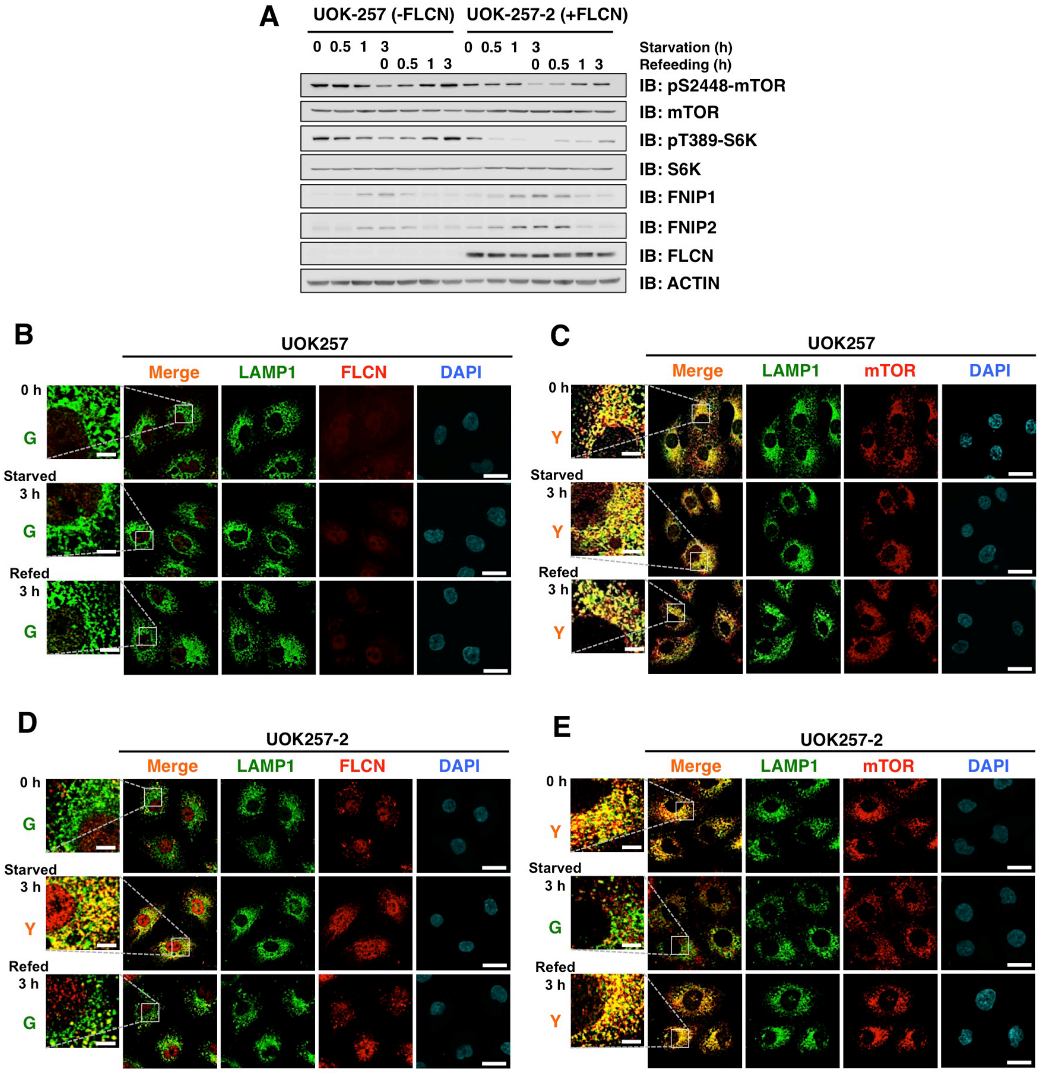

Supplementary Figure 3: The FLCN complex functions as a negative regulator of mTORC1 signaling in UOK-257 cells. A. Immunoblot (IB) analysis of whole cell lysates (WCL) derived from UOK-257 and UOK-257-2 cells. After being serum and amino acid-starved for 3 h, cells were treated with fresh 10% FBS DMEM and harvested at the indicated time points. **B-E.** Confocal images of UOK-257 (B and C) or UOK-257-2 (D and E) cells. DAPI-loaded HeLa cells were analyzed for co-localization of FLCN (B and D) (red) or mTOR (C and E) (red) with a lysosomal marker LAMP1 (green). Y (yellow) indicates predominant localization of FLCN or mTOR in the lysosome. Scale bars, 20 μm (5 μm in the enlarged images).

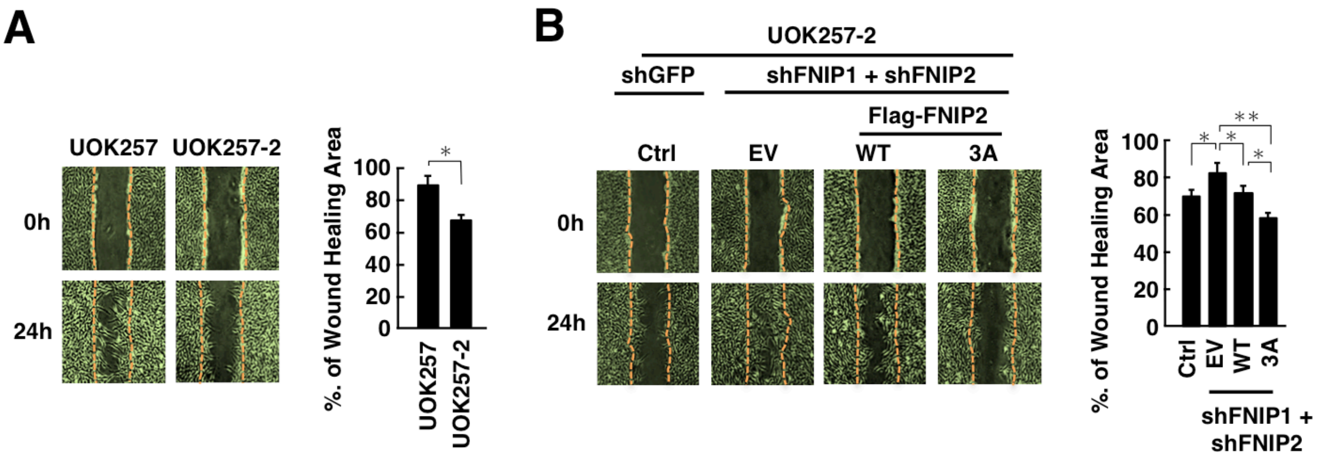

Supplementary Figure 4: Non-degradable FNIP2 mutant suppresses the motility of UOK-257-2 cells. A. UOK-257 or UOK-257-2 cells were plated for *in vitro* wound healing assays (left panel). Results were quantified and presented as means ± SD (right panel), n = 3, * $p < 0.05$. **B.** UOK257-2 cells presented in Figure 7A and 7C were plated for *in vitro* wound healing assays (left panel). Results were quantified and presented as means ± SD (right panel), n = 3, * $p < 0.05$, ** $p < 0.01$.
